# Unveiling practical insights of eHealth implementation in Europe: a grey literature review on legal, ethical, financial, and technological (LEFT) considerations

**DOI:** 10.3389/fdgth.2025.1575620

**Published:** 2025-08-14

**Authors:** B. E. Bente, N. Beerlage-de Jong, R. M. Verdaasdonk, J. E. W. C. van Gemert-Pijnen

**Affiliations:** ^1^ Centre for eHealth and Wellbeing Research, Section of Psychology, Health and Technology, Faculty of Behavioural, Management and Social Sciences, University of Twente, Enschede, Netherlands; ^2^ Section of Health, Technology and Implementation, Technical Medical Centre, University of Twente, Enschede, Netherlands

**Keywords:** implementation, eHealth, digital health, healthcare, legal, ethical, business model, stakeholder involvement

## Abstract

**Background:**

The implementation of eHealth technologies can improve healthcare efficiency, accessibility, and affordability. However, it involves complex legal, ethical, financial, and technological (LEFT) challenges that can impede success. While our previous scoping review identified barriers such as balancing compliance with innovation, funding gaps, and unclear business models, there remains a significant gap in understanding how these challenges manifest in real-world settings. This study uses grey literature to explore practical experiences and strategies in addressing LEFT challenges during eHealth implementation.

**Objective:**

This study aims to explore real-world experiences and perspectives on the legal, ethical, financial, and technological (LEFT) challenges encountered during eHealth implementation.

**Methods:**

A grey literature review was conducted by querying databases BASE and Policy Commons, consulting expert references for relevant reports, and using snowball sampling to identify additional relevant grey literature.

**Results:**

While the aim of this study was to explore practical experiences, the grey literature mainly reflects policy-level concerns, including strategic and regulatory challenges, with limited insight into how organizations navigate eHealth implementation in practice. Legal barriers include navigating complex regulatory frameworks, interpreting regulations, and concerns about data privacy. Facilitators focus on centralized governance and Europe's role in the global data market. Ethical barriers address inequalities in access, while facilitators emphasize patient autonomy, clear consent processes, and digital literacy. Financial barriers stem from inadequate funding structures and unclear financial requirements, with public-private partnerships as facilitators. Technological barriers revolve around interoperability issues due to national IT infrastructure limitations, with facilitators working to improve data exchange.

**Conclusions:**

This study highlights a disconnect between the strategic focus of available grey literature and the need for actionable, practice-based insights. The limited presence of real-world implementation experiences underscores the necessity for more operational documentation to support stakeholders facing interrelated LEFT barriers. Key challenges include the need for actionable legal and ethical frameworks, clearer ethical discussions aligned with legal requirements, sustainable financial infrastructures, and enhanced stakeholder involvement to address interoperability challenges. These challenges require cross-sector investment in IT infrastructures, harmonized data standards, and stronger collaboration among stakeholders. Coordinated efforts across all LEFT domains are crucial for effective eHealth implementation.

## 1 Introduction

The domain of eHealth is dynamic, driven by technological advancements and the increasing integration of digital healthcare technologies into practice. The presumed added value of eHealth lies in its potential to improve healthcare efficiency, accessibility, affordability, and patient outcomes, making it crucial to better implement these technologies to fully realize their benefits (1). Achieving optimal impact from these technological advancements and their integration, however, requires more than just innovation—it depends on successful and sustainable implementation. Yet, this often proves to be a significant challenge in practice. Recent discussions have increasingly focused on eHealth implementation, but these are typically framed from an organizational or eHealth development perspective, leaving legal, ethical, financial, and technological (LEFT) considerations underexplored or underestimated. Our prior scoping review (2) addressed these gaps by exploring the LEFT factors in implementing complex eHealth technologies, identifying barriers and facilitators inherent in this multifaceted domain. This exploration yielded insights into critical challenges for successful implementation, namely:

• **Balancing Compliance with Legal and Ethical Regulations while Fostering Innovation in eHealth Implementation.** The key challenges lie in ensuring compliance with stringent legal and ethical regulations (such as secure data management) without stifling innovation in eHealth development and implementation.
• **The Self-Perpetuating Cycle: Validity and Funding in the Transition from eHealth Research to Implementation.** A major challenge is breaking the cycle in which securing funding depends on demonstrating effectiveness, while gathering sufficient evidence of effectiveness often requires prior funding.
• **Navigating Uncertainties in Responsibility and Accountability in eHealth Implementation.** A critical challenge is the lack of clarity in regulatory and ethical frameworks regarding who is responsible and accountable for various aspects of eHealth implementation and daily use of the technology.
• **Business Modeling Gaps and Lack of Reimbursement Mechanisms for Financial Sustainability in eHealth Implementation.** A persistent challenge is the absence of clear reimbursement structures and sustainable business models, making it difficult to secure long-term financial viability for eHealth solutions.

These challenges underscore the complexities of eHealth implementation and emphasize the need for targeted strategies in legal compliance, funding, accountability, and financial planning. However, LEFT considerations are just one component of a broader set of factors that influence the success or failure of eHealth initiatives. Many other practical, contextual, and interpersonal elements also play a crucial role. Despite increased academic attention to these challenges, a significant knowledge gap remains: the “real-world”, practice-based experiences of organizations and stakeholders implementing eHealth technologies. To address this gap, this study conducts a comprehensive review of grey literature, including policy papers and other non-peer-reviewed sources. Grey literature offers valuable insights into recent developments, practical applications, and implementation barriers. These sources often contain actionable recommendations, reflecting how organizations interpret and apply multi-interpretable regulations to meet real-world needs (3, 4).

For example, government institutions and professional societies produce reports with guides, summaries and implementation tips for navigating implementation challenges (5). Additionally, consulting firms frequently release whitepapers to promote their services, offering insights into the difficulties faced by their clients. The quality of these reports varies; while some provide valuable guidance, others remain too general to provide concrete solutions for specific cases (6). Nevertheless, even in the absence of rigorous peer review, these documents provide valuable real-world insights from those involved in the actual implementation process, thus offering practical perspectives that are highly relevant for this study. Given the distinct legal and regulatory environments governing eHealth across continents, the focus of this study is, specifically on the European context. The European Union (EU) provides a unified framework through its Digital Health Strategy, making it an important reference point for understanding the LEFT-related challenges within Europe. This study ensures contextual accuracy by analyzing region-specific challenges, such as compliance with the General Data Protection Regulation (GDPR) (7) and the evolving European Health Data Space (EHDS) (8).

By delving deeper into the key challenges emerging from the barriers and facilitators identified in the previous study, this study aims to explore real-world experiences and perspectives that highlight these LEFT complexities during the implementation of eHealth technologies. Our goal is to uncover actual implementation experiences of healthcare organizations and companies, particularly through whitepapers, policy briefs, and similar documents, in which involved stakeholders (e.g., healthcare workers, researchers, developers, and policymakers) share their challenges, the measures they implemented to address these challenges, and how these efforts were realized in practice. By complementing the findings from academic literature (the previous scoping review) with insights from grey literature, this study bridges theoretical frameworks and practical experiences (3). Rather than making a direct comparison, the inclusion of grey literature enriches scientific findings by revealing how these frameworks are applied in practice. This approach supports the development of more effective and sustainable strategies to address real-world LEFT challenges in eHealth implementation in a comprehensive and balanced manner.

## 2 Methods

This grey literature adheres as closely as possible to the PRISMA-ScR (Preferred Reporting Items for Systematic Reviews and Meta analyses extension for Scoping Review) checklist (9), with specific adaptations as outlined in this section, acknowledging that grey literature necessitates a different approach than traditional systematic reviews. This review process was designed by a multidisciplinary team of researchers with expertise in eHealth and a data information specialist, focusing on various aspects of implementation.

### 2.1 Eligibility criteria

In alignment with the methodology established in our previous scoping review (2), Table 1 outlines the eligibility criteria for the current grey literature study. Selected documents must be disseminated by entities actively involved in the development and implementation of health technology, specifically focusing on eHealth technologies that facilitate the exchange of data between users and/or IT systems. Included documents must address one or more implementation aspects, encompassing legal, ethical, financial, or technological considerations. To ensure geographical relevance and comparability, studies focusing on non-European contexts are excluded. Scientific peer-reviewed works are intentionally excluded, as they have been comprehensively examined in our previous scoping review. Considering the fast-paced environment of eHealth development and the accompanying legal and ethical regulations, only documents published from 2018 onwards are included. Additionally, documents not available in English or Dutch, as well as newsletters, news releases, memorandums, and legislation, are excluded to uphold the integrity of our study. Initially, the eligibility criteria were assessed on a selection of documents, refining, and adjusting them as necessary based on these preliminary evaluations.

**Table 1:** Grey literature review eligibility criteria.

Inclusion criteria	Exclusion criteria
Reports published by governmental organizations, consultancy companies, NGO's, white paper advisory boards, or other organizations involved in the development and/or implementation of health technology.	Papers and reports published outside Europe (i.e., the paper or report was written about a non-European context)
Must focus on eHealth technologies that facilitate health or treatment related data exchange between users and/or IT systems	Peer-reviewed work (i.e., peer-reviewed studies, conference proceedings, scientific books, theses)
Legal, ethical, financial, and technological aspects of implementation (one, or more of the aspects)	Published <2018
	Unavailable in English or Dutch
	Newsletters, news releases, memorandums, or legislation

### 2.2 Search strategies and information sources

A comprehensive and systematic grey literature search plan was developed to incorporate three different searching strategies: (1) literature databases, (2) expert references, and (3) snowball sampling. These strategies were chosen by insights from a prior grey literature review on digitalization in healthcare (10). An overview of the three search strategies with used sources is provided in Table 2.

**Table 2:** Information sources and searching strategies.

1. Literature databases	2. Expert references	3. Snowball sampling
BASE and Policy Commons	Papers and reports recommended by stakeholders (e.g., researchers, developers, or policy makers with expertise in the legal, ethical, financial, and/or technological aspects of the implementation of eHealth).	Backward and forward snowballing

#### 2.2.1 Search strategy 1: literature databases

A systematic search was conducted across the BASE and Policy Commons databases, which are recognized for their extensive collections of grey literature and recommended by our institution's information specialist. On March 18, 2024, reviewer BB conducted the initial search. A structured query, developed in collaboration with eHealth experts and an information specialist, utilized search terms derived from topics identified in the scoping review, encompassing LEFT topics, synonyms for implementation (including adoption and practice), synonyms for eHealth (including digital health, technology, device, telemedicine, and platform) and terms as experiences, strategies, guidelines, and updates. Due to the differing formats and requirements of each database, tailored search strings were created. For BASE, three separate queries focused on legal [(law OR privacy OR regulation OR security OR law OR certificat*) AND health AND (ehealth OR technology OR digital OR device OR telemedicine OR platform) AND (implement* OR adopt* OR practice) AND (advice OR guideline OR strateg* OR experience OR policy)], ethical [(ethic* OR validat* OR eviden* OR responsib* OR accountab*) AND health AND (eHealth OR technology OR electronic OR digital OR platform OR telemedicine) AND (implement* OR adopt OR practice) AND (advice OR guideline OR strateg* OR experience OR policy)], and financial [(financ* OR fund* OR “business model” OR reimbursement) AND health AND (eHealth OR technology OR electronic OR digital OR platform OR telemedicine) AND (implement* OR adopt OR practice) AND (advice OR guideline OR strateg* OR experience OR policy)] aspects. Technological aspects were considered covered within these three queries. In contrast, Policy Commons allowed for a consolidated search string [title:(technology OR platform OR digital OR eHealth OR telemedicine) AND title:health AND title:(implement OR practice OR strateg* OR policy OR advic* OR financ* OR “business model” OR ethic* OR legal OR law OR regulat* OR privacy OR security OR responsib* OR accountab* OR reimbursement OR fund*)], requiring the terms to appear in the title to narrow the results. Although NexisUni was initially included as database, difficulties in exporting the documents (*n* = 296), combined with an assessment of the potential documents indicating limited relevance to the study, led to its exclusion from the final search.

#### 2.2.2 Search strategy 2: expert references

Eventually, the research team engaged 45 experts from their professional networks and through snowballing, including researchers from universities and colleges specializing in healthcare and technology or ICT, policymakers, legal experts, business developers, employees from companies involved in technology development and implementation, and individuals working for NGOs and research and consultancy firms. A summary of the scoping review findings and the current study's objectives were shared, along with the request for relevant documents or referrals to knowledgeable contacts.

#### 2.2.3 Snowball sampling

Snowball sampling was employed to identify additional documents through the references of the documents obtained from strategies 1 and 2.

### 2.3 Selection of evidence

The process began with the documents gathered from strategy 1 (BASE and Policy Commons) being entered into ASReview, an AI tool for screening (11). However, due to the nature of grey literature, particularly the frequent absence of abstracts, ASReview proved unsuitable for further screening. Therefore, ASReview was only used to eliminate duplicates. Consequently, all documents underwent independent manual review, including title and abstract screening (BB, NBJ) and full text-screening (BB, NBJ, RV, LGP), adhering to the established eligibility criteria, with any conflicts resolved through consensus. In strategy 2, submitted documents and contact referrals were organized in an Excel sheet. Each submitted document was reviewed (BB) against the eligibility criteria to determine inclusion. Additionally, the reference list of all documents retrieved from both search strategies were searched for relevant titles published after 2018 (Search strategy 3: Snowball sampling), which were screened against the eligibility criteria as well.

### 2.4 Data extraction and charting

The same approach as in the previous scoping review was used for data extraction. General document characteristics were recorded, such as the author(s), year of publication, affiliated institution(s), document objectives, and discussed topics, were recorded. The same coding scheme from the scoping review was applied, in which barriers and facilitators were categorized per LEFT domain. The lead researcher (BB) reviewed all full texts and systematically extracted key points related to these domains. Relevant fragments were coded and summarized using the coding scheme. The identified barriers and facilitators were subsequently categorized into topics through an iterative axial and selective coding process, closely aligned with those identified in the scoping review. The data extraction form was continuously refined with input from the research team throughout the process.

## 3 Results

### 3.1 Document selection

**Figure 1:**
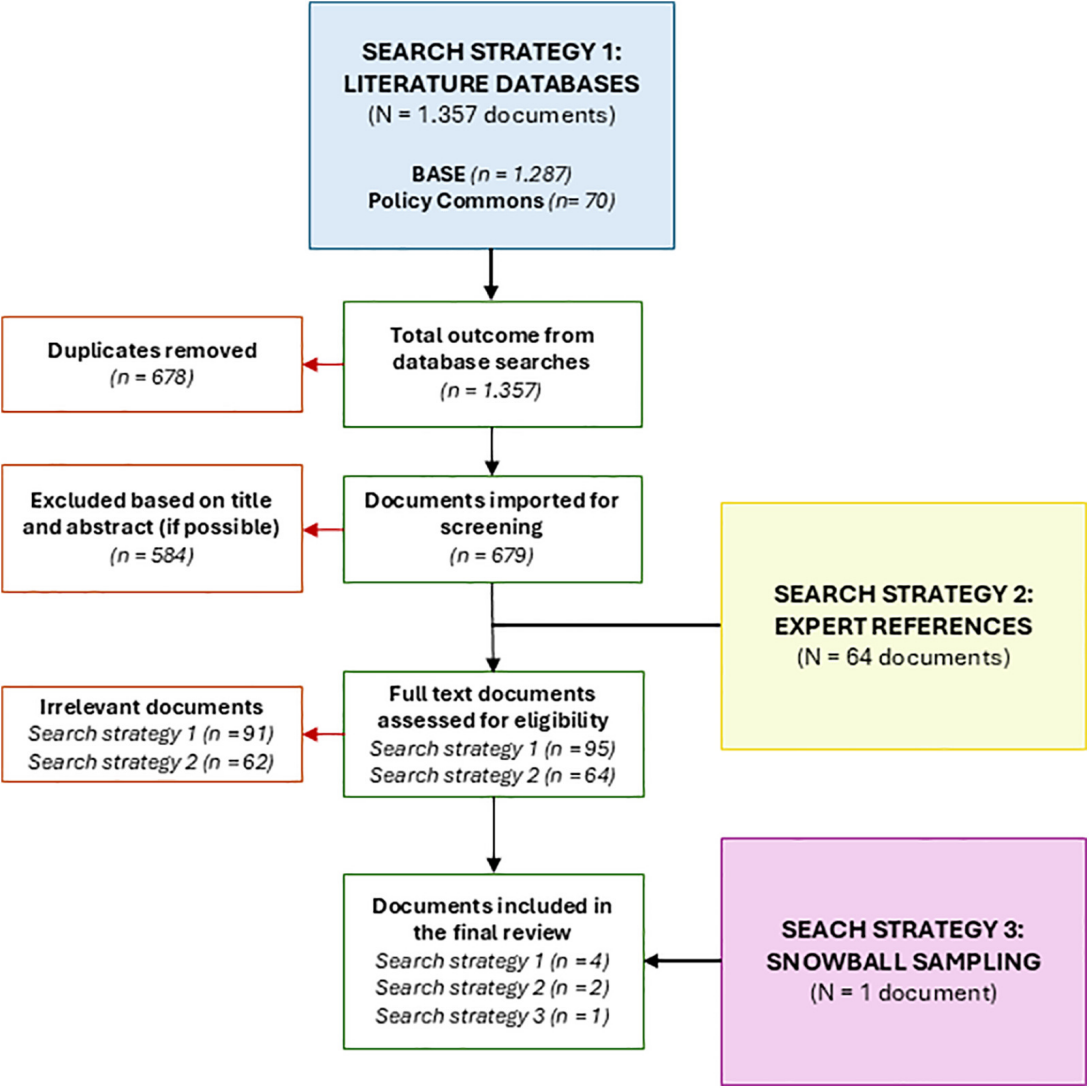
PRISMA (Preferred Reporting Items for Systematic Reviews and Meta-analyses) flowchart of included and excluded documents.

In search strategy 1 (literature databases), 1.357 potentially relevant documents were identified, published between 2018 and 2023. Following the removal of 678 duplicates (49.96%), 679 unique documents (50.04%) were subjected to assessment. This led to the eligibility of 95 (7.0%) full texts, of which ultimately, 4 documents (0.29%) were included. Search strategy 2 (expert references) rendered 64 potentially relevant documents of which ultimately 3 documents were included. Finally search strategy 3 rendered one additional document that was identified through snowball sampling and included in the final data extraction. The primary reasons for exclusion were non-European settings, peer-reviewed studies (as these were already covered in the formal scoping review), and insufficient information on the legal, ethical, financial, or technological aspects of implementation. See Figure 1 for a flowchart of the document selection.

### 3.2 Document characteristics

In total, this review included seven documents published between 2018 and 2023, representing diverse publication types, including reports, white papers, joint statements, and legal professional literature. Contributions come from various organizations such as the Nordic eHealth Research Network, Health Action International, and the Joint Research Centre of the European Union. These documents focus on a broad range of health technologies, particularly emphasizing the role of Artificial Intelligence (AI) in healthcare and digital health services. Additionally, some documents also focus on broader regulatory frameworks for health technologies rather than specific technologies. Topics covered encompass the ethical, legal, and social implications of AI technologies, regulatory frameworks for health data exchange, and strategies for digital health transformation across different European countries. The documents provide a comprehensive understanding of the current state of eHealth implementation, highlighting both opportunities and challenges for policymakers and stakeholders involved in the integration of innovative health technologies. In Table 3, an overview can be found of the included documents and their characteristics.

**Table 3:** Characteristics of the included documents.

#	Authors	Year	Title	Aim	Target group of publication	Health technology	Topics discussed
1	Nøhr et al. (16)	2020	Nordic eHealth Benchmarking. Towards evidence informed policies	To provide a foundation for benchmarking across the Nordic countries and support policymaking in the countries	National and international policy makers and scientific communities	eHealth (strategies) in general	LEGAL/ETHICAL/FINANCIAL/TECHNOLOGICAL: •eHealth strategies/policies in the Nordic •Cyber security in Nordic countries
2	Ake-Kob et al. (17)	2021	State of the art on ethical, legal, and social issues linked to audio- and video-based AAL solutions	To define the ethical and legal issues related to AAL technologies, review relevant legislation, study privacy perceptions influence by cultural and demographic factors, and investigate the benefits and barriers to adopting AAL technology for those in need of care.	Researchers and industrial partners from different fields (computing, engineering, healthcare, law, sociology) and other stakeholders (users, policymakers, public services)	Ambient Assisted Living (AAL) Technologies	LEGAL/ETHICAL: •Legal aspects in the context of AAL •Ethical aspects in the context of AAL •Societal challenges to video- and audio-based monitoring
3	van Kolfschooten (15)	2023	The Impact of Artificial Intelligence on Health Outcomes for Key Populations: Navigating Health Inequalities in the EU	To call for renewal of EU commitments and coordination of efforts of all EU institutions, Member States, and relevant stakeholders, with existing strategies, plans, and directives/guidelines/legislation, in concert with the general measures including several EU laws regarding liability, security, data protection, etc. (e.g., GDPR, MDR, Data Governance Act).	EU institutions, member states, and relevant stakeholders	Artificial Intelligence technologies in Medicine and Healthcare	LEGAL/ETHICAL/FINANCIAL/TECHNOLOGICAL •Concerns about health AI •Call for attention to both the positive and potential negative effects of health AI on key populations •Call for specific measures for health AI by EU organizations, Member States, and relevant stakeholders
4	Gomez-Gonzalez (18)	2020	Artificial Intelligence in Medicine and Healthcare: applications, availability, and societal impact	(1) To boost the EU's technological and industrial capacity and AI uptake across the economy, both by the private and public sectors, (2) To prepare for socio-economic changes brought about by AI, and (3) To ensure an appropriate ethical and legal framework	Policy developers, but also of interest for researchers studying the impact and the future of AI on healthcare, for scientific and technological stakeholders in this field, and for the general public	Artificial Intelligence technologies in Medicine and Healthcare	LEGAL/ETHICAL/TECHNOLOGICAL •A conclusive set of policy challenges •Social concerns and debates on AI in general press, social media, and other web-bases sources •Overview of AI technologies and their implementations, including perspectives, conflicting views, potential pitfalls
5	Wetenschappelijke Raad voor het Regeringsbeleid (14)	2021	Opgave AI. De nieuwe systeemtechnologie	Advies voor wetgeving rondom AI gebruik in Nederland	Addressed to the Dutch Prime Minister, Chair of the council of Ministers	Artificial Intelligence technologies	LEGAL: •Challenges for the implementation of AI
6	Dekker and Hooghiemstra (12)	2023	Europese ruimte voor gezondheidsgegevens (EHDS)	Answering the question: “To what extent does the EHDS influence the existing and future legal framework for health and data protection?"	Policymakers, healthcare professionals, and medical associations	Exchange of electronic personal health data for primary and secondary use	LEGAL/ETHICAL/FINANCIAL: •Addresses the frameworks proposed from Europe for the exchange of health data now that the European Commission has presented the “European Health Data Space” as a draft regulation. •Concerns regarding privacy, consent, and market competitiveness of Europe
7	Thiel et al. (13)	2018	#SmartHealthsystems International comparison of digital strategies	To evaluate the state of digitalization in healthcare systems across 17 countries, identify successful strategies and barriers, and provide recommendations to improve and accelerate the digital transformation of Germany's healthcare system	Policymakers, healthcare professional, and stakeholders involved in the digital transformation of healthcare systems.	Digital health services and strategies	LEGAL/ETHICAL/FINANCIAL/TECHNOLOGICAL: •Comparative analysis of digitalization of healthcare systems in 17 countries •Succes factors and barriers •Policy activity •Readiness of digitalization •Actual use of data

### 3.3 Barriers and facilitators of eHealth implementation

For each domain—legal, ethical, financial, and technological aspects of implementation—both barriers and facilitators have been identified, which will be discussed below in different sections. The lack of entries for certain barriers or facilitators in the tables does not indicate their absence in that domain; rather, it reflects that these were not identified in the reports included in this review.

#### 3.3.1 Legal aspects

Several barriers and facilitators were identified related to legal aspects of the implementation of eHealth. These factors focus on regulatory frameworks, political and institutional coordination, and legal boundaries and liability.

##### 3.3.1.1 Legal barriers

The complexity of regulations hinders navigation and effective collaboration (12). In particular, unclear and inconsistent regulations exacerbate this issue, making it challenging for stakeholders to interpret and comply with existing legal requirements (13). Moreover, early-stage technology regulation poses additional challenges, as uncertainties about its functioning and societal impacts delay the identification of necessary legal measures (14). Furthermore, privacy and data protection issues further complicate eHealth implementation (13, 15), with countries proactively addressing these challenges achieving greater success (13). Countries with less developed or absent legal frameworks (e.g., France and Portugal) experience more challenges in implementing digital health strategies, compared to those with comprehensive legal frameworks, which show higher implementation success (13). In addition, regional disparities in legal adaptation also hinder progress, although some countries (e.g., Italy), have managed to successfully maintain overlapping frameworks that support overall development (13).

The complexity of political and institutional structures—including policies, resource ownership, responsibilities for strategy execution, licensing requirements, and the involvement of multiple organizations—can further hinder implementation efforts (16). In contrast, countries lacking centralized political coordination (e.g., Germany and Spain) tend to experience lower implementation success than countries with dedicated coordination bodies (13). Finally, Europe faces competitive challenges in the global data market, particularly against dominant players like the US and China, which may sometimes place relatively less emphasis on fundamental patient rights, such as privacy, compared to European countries (12). To compete effectively, Europe must transition from a trade union to a data union, enabling a unified response to international developments (12). See Table 4 for an overview of all identified barriers of legal aspects.

**Table 4:** Overview of the identified **legal** barriers.

Topics	Barriers
Regulatory framework (*n* = 5 reports)	Complexity of legal framework makes it difficult to navigate (12)
	Lack of Regulatory Mechanisms for AI in healthcare (15)
	Unclear or inconsistent regulations (13)
	Lack of a legal framework (13)
	Difficult to Regulate in Early Stages (14)
	Privacy and Data protection risks (13, 15)
	Regional disparities in legal adaptations (13)
	Challenges with Medical Device Regulation (15, 17)
	Unclear Boundaries between Products and Services (17)
Political and Institutional Coordination (*n* = 3 reports)	Complexity of political and institutional structures (16)
	Lack of centralized political coordination (13)
	Competitive challenges for Europe in the Global Data Market (12)

##### 3.3.1.2 Legal facilitators

Despite the barriers outlined, several facilitators can enhance the implementation of eHealth. Primarily, ensuring data confidentiality and protection is fundamental (13, 15, 17, 18). For example, cybersecurity mechanisms (17) and safeguards against unethical use of Artificial Intelligence (AI), such as the commercial exploitation of personal health data, can improve outcomes and strengthen trust (15). In addition, a robust policy infrastructure and legal framework are also critical for success (13, 14). Prioritizing standardization and certification activities (12, 13), along with clear guidelines for managing these processes (12). Moreover, developing protocols for interoperability and security within the EHDS can facilitate successful implementation (12). Countries with strong cooperation on standards (e.g., Estonia and Denmark) tend to achieve greater success in their digital health efforts than those without (13). Similarly, implementing cybersecurity mechanisms is similarly crucial for strengthening the regulatory environment of eHealth (17). These practices, including secure communication, software updates, encryption, and vulnerability reporting mechanisms, safeguard eHealth systems and enhance data confidentiality and protection (17).

Furthermore, strategic involvement of stakeholders in policy development and alignment with eHealth strategies is essential for facilitating successful implementation (13, 16). Countries with greater political involvement and proactive government action in digital health initiatives (e.g., Estonia, Denmark, Sweden and Portugal) typically achieve more successful implementation (13). Finally, proactive government action on regulating surveillance activities, including the monitoring of data collection practices and the concentration of power among large companies, is also key (13, 14). See Table 5 for an overview of all identified facilitators of legal aspects.

**Table 5:** Overview of the identified **legal** facilitators.

Topics	Facilitators
Regulatory framework (*n* = 6 reports)	Ensure data confidentiality and protection (13, 15, 17, 18)
	Establish and maintain a robust policy infrastructure and legal frameworks (13, 14)
	Robust activities and clarity regarding standardization and certification (12, 13)
	Strengthening the market approval for medical devices using AI (15)
	Clarifying responsibilities for personal data processing (12, 13)
	Enhancing patient rights protection (12)
	Implement Cybersecurity Mechanisms (17)
	Ensuring medical confidentiality in secondary data use (12, 17)
Political and Institutional Coordination (*n* = 3 reports)	Strategic stakeholder involvement in policy and eHealth strategy alignment (13, 16)
	Government action on Surveillance and Data collection (13, 14)

#### 3.3.2 Ethical aspects

Several barriers and facilitators were identified related to ethical aspects of the implementation of eHealth. These factors focus on autonomy, consent, data ownership, risk assessment, stakeholder engagement, equity, and ethical frameworks.

##### 3.3.2.1 Ethical barriers

A significant barrier to successful eHealth implementation is the lack of efficient stakeholder involvement (13, 17). Key stakeholders include clinicians, patients, healthcare leaders, policymakers, IT service providers, and vendors of the eHealth systems. Countries with minimal stakeholder engagement, such as France and NHS England, often face challenges in implementing digital health strategies compared to those with more active participation from these stakeholders in eHealth development and implementation (13). Moreover, engagement in the development of digital health applications, legislative processes, and implementation strategies are mentioned as crucial for success. Another significant barrier is the inequality in access to technologies (15, 17, 18). For example, factors such as a lack of awareness, inadequate product designs that do not align with user or environmental preferences, low production quality, and financial constraints hinder equitable access to eHealth (17). Furthermore, AI technologies may worsen health inequalities by limiting access for individuals with insufficient digital literacy or those who do not meet accessibility requirements (15). Vulnerable populations, including racial and ethnic minorities, are particularly at risk of being disadvantaged by these technological disparities (15). In addition, while digital health advancements are expected to bring positive economic impacts, there is a need to address the risk of exacerbating existing inequalities, which calls for the development of new models for coverage, insurance, and affordability (18). Another barrier arises from the complexity of determining liability in multi-actor eHealth systems, where the involvement of multiple technologies and stakeholders makes it difficult to pinpoint the exact source of product failures or system malfunctions (i.e., determining who is liable for damages or failure of the technology) (17). See Table 6 for an overview of all identified barriers related to ethical aspects.

**Table 6:** Overview of the identified **ethical** barriers.

Topics	Barriers
Equity (*n* = 3 reports)	Inequalities in Access to eHealth Technologies (15, 17, 18)
Stakeholder engagement (*n* = 2 reports)	Low stakeholder involvement (13, 17)
Liability (*n* = 1 report)	Complex liability in eHealth (17)

##### 3.3.2.2 Ethical facilitators

Despite the barriers highlighted, several facilitators can support the successful implementation of eHealth by addressing ethical concerns. Empowering patient autonomy is essential for fostering trust and acceptance of eHealth technologies (13, 15–18). By providing users with all relevant information about the technology they interact with, users (e.g., patients) are enables to make informed decisions, which in turn fosters confidence in these systems (17). Related to this, involving users in decision-making processes, such as policy or strategy development, contributes to better implementation outcomes and higher adoption rates (13, 16, 18). Moreover, involving stakeholders throughout the entire development, implementation, and market access process through methods like ethical dialogue helps prevent, mitigate, or resolve ethical challenges that could harm users or hinder user adoption (15, 17). Furthermore, ensuring clear consent and data ownership is another essential facilitator (12, 17). While the EHDS allows patients to control access to their data by healthcare professionals, it lacks oversight concerning non-healthcare entities, such as suppliers, raising privacy concerns (12). In this context, clear guidelines and transparency regarding data ownership and consent processes can help mitigate these concerns and support the ethical use of data in eHealth (17).

A comprehensive evaluation of benefits, risks, and alternatives is essential for eHealth implementation (17). This assessment should address both current and potential future risks, which must be weighed against anticipated benefits. Inadequate evaluations could expose users to unforeseen risks or insufficient benefits, diminishing adoption and raising ethical concerns (17). In line with this, understanding who benefits, the duration of benefits, and the existence of better alternatives is critical for effective decision-making and broader technology uptake (17). Minimizing discrimination and bias is another important factor (15). Transparency about algorithms, assessing bias impacts, and maintaining data quality are essential to minimize these challenges (15). Additionally, the implications of new technologies should be considered not only for patients but also for healthcare professionals, as new technologies can affect job roles, quality control, training, and relationships between patients and employers (18). Investing in digital literacy is a facilitator in promoting equity (15). By enhancing digital literacy education, both healthcare professionals and patients gain the skills necessary to navigate eHealth technologies effectively, fostering inclusivity and improving equitable access (15).

Finally, the incorporation of ethical frameworks into (national) digital health policies is a vital facilitator (13). Countries that have adopted such frameworks, like Denmark, demonstrate greater success in eHealth implementation compared to those without such ethical guidelines (13). However, it is essential to regularly update these frameworks—both ethical and legal—especially as rapid technological advancements can outpace existing regulations, such as the Medical Device Regulation (MDR), leading to potential harms or adoption challenges if existing laws are not adapted in time or new regulations are not introduced. See Table 7 for an overview of all identified facilitators related to ethical aspects.

**Table 7:** Overview of the identified **ethical** facilitators.

Topics	Facilitators
Autonomy, Consent, and Data ownership (*n* = 6 reports)	Supporting Patient Autonomy and Empowerment (13, 15–18)
	Providing Clear Consent and Ownership for Data Use (12, 17)
Risk, Impact, and Bias assessment (*n* = 3 reports)	Assessment of eHealth benefits, risks, and alternatives (17)
	Minimize risks of discrimination and bias (15)
	Consider the impact on healthcare professionals (18)
Stakeholder engagement (*n* = 4 reports)	Involve stakeholders in the development and implementation of technology (13, 15–17)
Equity (*n* = 1 reports)	Investing in Digital Literacy for eHealth (15)
Ethical frameworks and policies (*n* = 1 report)	Incorporate ethical frameworks in digital health policies (13)

#### 3.3.3 Financial aspects

Several barriers and facilitators were identified related to financial aspects of the implementation of eHealth. These factors focus on financial structures and resource allocation.

##### 3.3.3.1 Financial barriers

A significant barrier to the successful implementation of eHealth is the misalignment between private sector incentives and healthcare goals (15). In particular, profit-driven development often prioritizes commercially viable populations, neglecting actual patient needs, which hampers alignment with broader healthcare objectives (15). Additionally, weak incentives for private-sector cooperation limit the formation of essential partnerships for sustainable eHealth implementation, reducing engagement from key private entities (13). Moreover, according to Thiel et al. (13), countries with insufficient financial investment in infrastructure and system development (e.g., Germany and Poland) face challenges in implementing digital health strategies effectively. These financial gaps result in poor outcomes for eHealth implementation, particularly in countries that allocate limited resources to digital health initiatives (13). See Table 8 for an overview of all identified barriers related to financial aspects.

**Table 8:** Overview of the identified **financial** barriers.

Topics	Barriers
Financial structures and Resource Allocation (*n* = 2 reports)	Private, Profit-driven Development of eHealth (15)
	Financial gaps in infrastructure and system development (13)
	Low financial allocation for digital health (13)
	Weak incentives for private-sector cooperation (13)

##### 3.3.3.2 Financial facilitators

Several facilitators can enhance the successful implementation of eHealth, beginning with ensuring financial clarity (12, 13, 16). This includes visualizing the economic benefits of eHealth (14) and clarifying the costs related to patients' free access to these technologies (12). Countries with strong financial support and well-defined arrangements, often from governmental sources or public-private partnerships (e.g., in countries such as Denmark and Belgium), typically achieve better outcomes on implementation success indices (13). Furthermore, aligning public investments with national health goals enhances the effectiveness of eHealth initiatives (13). Additionally, addressing existing financial gaps and optimizing resource allocation is crucial to improving implementation success (13). Finally, established public-private partnerships for funding projects can enhance both resource availability and project viability, contributing to sustainable eHealth development (13). See Table 9 for an overview of all identified facilitators related to financial aspects.

**Table 9:** Overview of the identified **financial** facilitators.

Topics	Facilitators
Financial structures and Resource Allocation (*n* = 3 reports)	Ensuring financial clarity and incentives (12, 13, 16)
	Addressing financial gaps and resource allocation (13)
	Established public-private cooperation in funding projects (13)

#### 3.3.4 Technological aspects

Several barriers and facilitators were identified related to technological aspects of the implementation of eHealth. These factors focus on data integrity, integration and interoperability, and technical infrastructures and resources. See Table 10 for an overview of all identified barriers and facilitators related to technological aspects.

**Table 10:** Overview of the identified **technological** barriers.

Topics	Barriers
Data integrity (*n* = 2 reports)	Errors in data (15, 18)
Interoperability (*n* = 2 reports)	Lack of interoperability (12, 13)
Technical infrastructure and resources (*n* = 1 report)	Limited technical resources (13)
	Poor IT infrastructure (13)

##### 3.3.4.1 Technological barriers

Several technological barriers hinder the successful implementation of eHealth. For example, errors in datasets, especially those used for AI, can introduce bias, and systems may process inaccurate data without issuing warnings, which can lead to flawed results (15, 18). Another significant barrier is the lack of interoperability among systems (12, 13). In European countries, there is a strong demand for harmonization of electronic health data, covering both primary and secondary use, particularly in terms of semantic interoperability (12). This process of harmonization leads to standardization, ensuring consistent labeling and meaning of data across different systems. Without such harmonization and standardization, countries face significant interoperability challenges, preventing them from realizing the full potential of their eHealth systems (13). This issue is exacerbated when different regions, organizations, or sectors (e.g., inpatient and outpatient care) rely on incompatible systems, significantly limiting data exchange (13). Additionally, the limitation of technical resources further hinders successful eHealth implementation (13). Many countries struggle with inadequate IT infrastructure, which prevents the effective implementation of digital health initiatives (13). See Table 4 for an overview of all identified barriers related to technological aspects.

##### 3.3.4.2 Technological facilitators

Several technological facilitators can promote the successful implementation of eHealth. Enhancing data transparency and availability is crucial for maintaining data integrity (15, 16, 18). For example, requiring the registration of all health technologies, especially those utilizing AI, in the EU public database can significantly improve transparency (15). Insufficient transparency regarding data availability jeopardizes stakeholder trust and undermines the overall effectiveness of eHealth initiatives (16). Furthermore, mitigating data bias and errors is essential for maintaining reliability. Ongoing efforts to improve dataset quality are particularly critical for AI applications, as poor data input can have severe consequences (15). Another facilitator is the development of robust technical infrastructure (13). For example, countries like Estonia and Israel demonstrate how a well-established national infrastructure can facilitate the integration of digital healthcare services and provide comprehensive access to patient data across different organizations (13). Additionally, countries with well-integrated, interoperable electronic health records (EHR) systems generally achieve higher performance in eHealth implementation (13). See Table 11 for an overview of all identified facilitators related to technological aspects.

**Table 11:** Overview of the identified **technological** facilitators.

Topics	Facilitators
Data integrity (*n* = 4 reports)	Enhance data transparency and availability (15, 16, 18)
	Mitigate data bias and errors (13, 15, 18)
	Improving quality of datasets (15)
Technical infrastructure and resources (*n* = 1 report)	Ensuring robust technical infrastructure (13)

## 4 Discussion

### 4.1 Principal findings

This study aimed to explore real-world experiences and perspectives on the legal, ethical, financial, and technological (LEFT) challenges encountered during the implementation of eHealth technologies.

• **Legal aspects:** The grey literature highlighted challenges related to navigating complex legal frameworks (e.g., identifying, understanding, and interpreting the applicable regulations). It also pointed to difficulties in applying these regulations correctly, alongside concerns about data privacy, protection, and confidentiality. Additionally, the grey literature emphasized political and institutional structures, such as the need for centralized governance and Europe's position in the global data market.
• **Ethical aspects:** Ethical considerations in the grey literature primarily focused on consent, patient autonomy, and data transparency. However, the grey literature primarily presented high-level policy statements without providing concrete practical details or specific arguments. Notably, the validation of eHealth technologies was scarcely addressed in the grey literature.
• **Financial aspects:** Grey literature acknowledged the importance of public-private collaboration but lacked details on business model strategies, reimbursement mechanisms, and financial sustainability. It also identified barriers such as misalignment between private-sector incentives and healthcare goals, as well as insufficient infrastructure investment.
• **Technological aspects:** Interoperability challenges were frequently mentioned in grey literature, particularly in the context of national infrastructure limitations. However, practical solutions for overcoming these barriers remained underexplored.

Overall, the grey literature primarily captured challenges at a higher policy level rather than concrete experiences from companies or organizations implementing eHealth technologies. While we aimed to also include documented cases—such as whitepapers—detailing how LEFT challenges during implementation were addressed and encountered, the encountered documents largely focused on broader, high-level strategic and regulatory discussions. These discussions were often centered around policy and governance rather than specific practical challenges faced by implementers. As a result, insights in practical mitigation strategies, as well as firsthand experiences with LEFT implementation challenges and the solutions applied in daily practice to overcome them, remained scarce, particularly in the ethical and financial domains. The gap between theoretical frameworks and practice-based evidence persists, particularly in areas such as balancing legal and ethical compliance with innovation (where regulatory rules can sometimes hinder the development and adoption of innovative solutions), clarifying the responsibility and accountability, and addressing financial sustainability through viable business models and reimbursement strategies.

### 4.2 Reflection

#### 4.2.1 Combining insights from grey literature and previous scoping review

This current study complements our previous scoping review by providing additional policy- and practical insights into the legal, ethical, financial, and technological (LEFT) aspects of eHealth implementation. While the scoping review offered more detailed insights, grey literature captured recent policy discussions and localized applications. However, as highlighted in Section 4.1, grey literature still often remained at a high-level strategic discourse, with limited practical solutions or tested implementations, potentially reflecting the difficulty of addressing these challenges in real-world settings. Below, the findings from both the scoping review and grey literature study are compared and reflected upon across the LEFT domains.

##### 4.2.1.1 Legal aspects

Both the scoping review and grey literature identified the complexity of regulatory frameworks, often describes as unclear and inconsistent, particularly in terms of how various regulations are applied across different EU members. This complexity arises from varying interpretations and implementations of key concepts, such as patient consent, data access, and data (re-)use. For example, discrepancies exist in how different countries define and handle patient consent, as well as the access and reuse of health data. These discrepancies include differences in consent models, data protection requirements, and the scope of permissible data usage across borders. This fragmentation poses significant challenges for consistent and interoperable eHealth implementation. The European Health Data Space (EHDS) is expected to address some of these issues, but its precise impact on data reuse and governance will depend on clear national-level data policies (19).

Moreover, the political and institutional structures are becoming increasingly important for eHealth implementation. Both the scoping review and grey literature highlight the need for centralized governance to reduce fragmentation and improve interoperability. Such centralized governance structures are especially critical at the local level, where there are calls for stronger institutional support to address regulatory inconsistencies and ensure smoother collaboration. The grey literature further emphasized that without these centralized structures, local challenges will remain unaddressed, revealing a “cry for help” from practice, where the real-world difficulties of navigating fragmented regulations are felt most. This urgency underscores the need for cohesive governance that bridges gaps between different national regulations and adoption of techniques for safe and secure data sharing across borders (20, 21).

In addition, the MDR acts as both a facilitator and a barrier. While it enhances safety, reliability and transparency, its stringent requirements can stifle innovation, especially for smaller entities with limited financial resources and capacity to meet the demands of providing a device's effectivity. Consequently, many European start-ups have opted to obtain certification through the Food and Drug Administration (FDA) in the USA, resulting in substantial economic impact in Europe (22). Another unintended consequence has been a shortage of certain medical devices (“orphan” devices) in hospitals, as companies chose not to recertify these due to high costs and limited profitability (23). Acknowledging these negative impacts, the European Commission has recently introduced measures to mitigate these effects (24). They proclaimed calls to investigate the issues and stressed the importance of the market itself taken action. However, it will likely take a long time before results will become noticeable. This dual role of the MDR—ensuring safety and transparency while simultaneously imposing barriers to innovation—underscores the need for a balanced approach. Therefore, clear harmonized legal frameworks are crucial for overcoming fragmentation and improving interoperability in eHealth systems.

##### 4.2.1.2 Ethical aspects

Ethical challenges, especially around responsibility, accountability, and data consent, remain a key barrier to eHealth implementation. One major issue highlighted in the scoping review is the lack of clear regulatory and ethical frameworks to define stakeholder roles and responsibilities, leading to uncertainty during the implementation and everyday usage of eHealth technologies. While the importance of ethical principles is recognized, these challenges are rarely addressed in the grey literature, often overlooking the practical aspects of real-world implementation challenges on the work floor. This highlights a disconnect between scientific knowledge and real-world practice, underscoring the need for clear ethical guidelines—particularly regarding data re-use and accountability— which closely overlap with the previously discussed legal aspects (e.g., GDPR) that govern data protection and regulatory compliance. Ensuring alignment between ethical and legal frameworks is crucial for eHealth technologies to be considered legitimate and trustworthy by society.

##### 4.2.1.3 Financial aspects

Financial sustainability remains a significant barrier to eHealth implementation. While both studies acknowledged the importance of public-private collaboration, the scoping review offered specific insights into business model strategies, reimbursement mechanisms, and financial sustainability—topics largely underexplored in grey literature. The lack of adequate funding, combined with insufficient clinical evidence, creates a cycle that hinders progress (2). Europe faces a competitive disadvantage in the global data market, particularly compared to regions like the United States and China (12). This disadvantage is further exacerbated by the earlier discussed trend of European start-ups seeking certification and market entry in the USA due to high costs and complex requirements of MDR compliance (22). These dynamics not only lead to economic losses but also highlights the pressing need for Europe to strengthen its position through strategic investments and the development of financially viable business models that align with global market demands and foster competitiveness. To overcome these barriers, Europe must focus on developing innovative reimbursement policies, subsidies, and public-private collaborations (25). Rather than being solely a topic for scientific research, developing sustainable financial infrastructures for eHealth requires a coordinated effort involving multiple stakeholders, including policymakers, healthcare providers, industry partners, and researchers. Academic research can play a key role by providing evidence-based insights that inform practical financial strategies, ensuring they align with both industry needs and evolving policy frameworks. To strengthen Europe's competitive position in the eHealth sector, it is crucial to develop business models that are not only viable within the European regulatory framework but also adaptable to global market demands. This requires the establishment of long-term financial mechanisms –such as reimbursement structures and funding programs—that facilitate the scalability and sustainability of eHealth initiatives.

##### 4.2.1.4 Technological aspects

Interoperability remains a fundamental challenge across all domains, particularly in the technological domain. Both studies recognized this issue, but with different emphases: grey literature primarily focused on national infrastructure limitations, such as insufficient harmonization of electronic health data and fragmentation between inpatient and outpatient systems, while the scoping review addressed software and hardware- related barriers, such as issues with data connectivity between systems in healthcare organizations and incompatibility of EHR. Additionally, the scoping review provided more detailed information on security frameworks and integration complexities. Fragmented IT infrastructures, inconsistent data standards, and political decisions—such as the blocking of a centralized electronic health record (EHR) system (26) –hinder seamless health data exchange. These interoperability issues delay the efficient use of eHealth technologies and exacerbate challenges related to data quality, trust, and cross-institutional collaboration. Effective data governance is crucial to address these challenges. Maximizing the potential of eHealth requires smarter data re-use, which depends on both technical solutions and cultural shifts. Within the European Union (UN) and European Economic Area (EEA), the GDPR sets clear boundaries for processing personal data, safeguarding individuals' privacy rights (7). Privacy enhancing Techniques, such as encryption of high-sensitive data, allow re-use of data use without GDPR constraints (27). However, legal mechanisms alone are insufficient for realizing data-driven healthcare. Effective data sharing and validation require strong multi-stakeholders' collaboration to create trust and agreements on (re-)use of data across organizations, especially in the context of AI-driven technologies (21).

The increasing reliance on AI in eHealth technologies further compounds these challenges. The “black box” nature of many AI systems raises concerns about bias in decision-making, particularly regarding gender, ethnicity, and age (28). Ensuring fairness, reliability, and transparency throughout an AI system's lifecycle is crucial for fostering trust in both the technology and the governance frameworks that support it. As AI becomes more integrated into healthcare technologies, addressing these concerns is critical to ensuring equitable decision-making and mitigating bias in health data use.

Trust and willingness to share data are essential to adopt privacy-enhancing technologies (PETs) (21). Stakeholder confidence in PETs' ability to protect privacy and ensure ethical data use is essential, as is trust in governance systems to prevent misuse. A lack of trust—often rooted in limited knowledge and concerns of losing control of sensitive data—hinders data sharing. Furthermore, unclear data semantics and insufficient interoperability further complicate cross-institutional collaboration (21). Overcoming these barriers requires coordinated efforts among medical, ethical, financial, and technical stakeholders.

### 4.3 Recommendations

A key observation in this study is that while grey literature provided valuable supplementary insights, it did not resolve the significant key challenges identified in our previous scoping review which served as the starting point for this study. While we hoped the grey literature would provide solutions from practice or experiences (e.g., from companies or governments) to address these challenges, it did not offer actionable information. This suggests that, although the field recognizes these profound challenges, it has yet to develop concrete, practical solutions to address them effectively. Therefore, the recommendations presented below aim to further investigate and explore potential approaches to these challenges, with the intention of contributing to more informed and practical strategies for advancing eHealth implementation.

Several frameworks designed to address eHealth implementation challenges [e.g., step-by-step guides or checklists designed to provide structured guidance for implementation of eHealth (29, 30)] were considered for this study but were ultimately excluded because they did not adequately address the critical LEFT aspects. While these frameworks raise awareness, they may not have been designed to offer detailed guidance, as they did not explore or provide actionable strategies for overcoming the complexities within LEFT domains (31). This gap is especially concerning as eHealth transitions from a standalone tool to an integrated digital health and data infrastructure. Existing toolkits and frameworks have yet to catch up with this transition, and without clear ownership and coordinated efforts, progress will remain slow (32). To prevent this, we propose the following recommendations based on the findings from both the scoping review and grey literature.

• **Alignment between Ethical and Legal Frameworks.** To successfully implement eHealth technologies, there needs to be a clear and coherent alignment between ethical and legal frameworks. The current lack of guidance on how ethical principles should be integrated into legal structures creates inconsistencies in compliance and regulation. Stakeholders must establish mechanisms to clarify these regulations and promote a harmonized approach that reduces uncertainty, and fosters trust among healthcare providers, developers, and patients. This requires coordinated governance at the national and EU level to ensure consistency in patient consent, data access, data (re-)use, and cross border interoperability, while also addressing regulatory inconsistencies and improving collaboration. Additionally, clear ethical guidelines—particularly regarding data (re-)use, consent, responsibility, and accountability—must be embedded within these frameworks to enhance trust and legitimacy in eHealth technologies.
• **Balancing Regulatory Compliance and Innovation.** While regulatory frameworks are essential to ensure safety and accountability, overly rigid regulations can hinder the development and adoption of novel eHealth solutions. A more flexible and risk-based regulatory approach is needed, to differentiate between low- and high-risk innovations, ensuring that compliance efforts are proportionate to potential risks. This includes exploring potential adjustments to the Medical Device Regulation (MDR) to better support smaller entities and improve the availability of “orphan” medical devices, while maintaining safety and transparency. Additionally, regulatory pilot programs and iterative compliance models can provide controlled environments for testing new technologies, allowing for real-world validation without prematurely restricting innovation.
• **Ensuring Sustainable Financial Models.** A major challenge in eHealth implementation is the lack of sustainable financial infrastructures. Many funding mechanisms remain fragmented and short-term, creating uncertainty for long-term adoption. To address this, a multi-pronged approach is needed that combines scalable investment strategies, reimbursement policies, and value-based financing. Governments and investors should explore sustainable reimbursement models that incentivize high-quality, cost-effective digital health solutions, while also ensuring financial feasibility for smaller innovators. Public-private partnerships can play a key role in securing long-term funding, particularly for innovations that improve interoperability and system efficiency. Additionally, value-based financing—where financial incentives are linked to patient outcomes and overall system efficiency—can help shift investments towards impactful and cost-effective eHealth solutions. Embedding cost-sharing mechanisms into eHealth business models can further enhance financial transparency and scalability, ensuring that the financial burden is distributed among relevant stakeholders. Actively involving key stakeholders in decision-making processes fosters trust and buy-in, increasing the likelihood of sustainable financial support and long-term adoption.
• **Achieving Interoperability to Enable Seamless Data Exchange.** Interoperability is not just a technical issue—it requires coordinated efforts across medical, ethical, financial, and technological domains. Poor interoperability hinders data exchange, disrupts continuity of care, and limits the integration of eHealth technologies into healthcare systems. While initiatives such as the EHDS aim to standardize data governance and improve accessibility, further efforts are required to address implementation challenges and ensure seamless cross-border collaboration. In particular, harmonized data standards must be effectively adopted and enforced to improve data quality and enable cross-institutional collaboration. Additionally, governance frameworks should not only balance privacy protection with data utility but also provide clear, actionable guidance on how to operationalize secure and accessible data sharing in practice. Stronger multidisciplinary collaboration among healthcare providers, technology developers, and policymakers is needed to align regulatory, technical, and operational requirements, reducing fragmentation. Finally, fostering trust in Privacy-Enhancing Technologies (PETs) through real-world case studies and transparent communication can help demonstrate the socio-economic benefits of secure and interoperable eHealth technologies.

An overarching theme in all of these recommendations is the critical role of stakeholder involvement in eHealth implementation. Encouraging shared decision-making among stakeholders (e.g., developers, users, and investors) helps address complex LEFT challenges by ensuring diverse perspectives are reflected. Early and continuous engagement of stakeholders is essential to align eHealth technologies with user needs and policy objectives. Fostering cross-sector partnerships—across healthcare, technology, legal, and business sectors—helps overcome siloed approaches, promoting innovation, and enabling a more holistic, sustainable approach. Additionally, building a culture of trust and transparency among stakeholders is also crucial to address resistance, improve collaboration, and ensure all parties are accountable to the shared goals of eHealth implementation.

As a next step, to support stakeholders—including eHealth developers, researchers, policymakers, legal experts, and business leaders—in navigating the complex LEFT challenges of eHealth implementation, we are developing a LEFT roadmap (33). This structured framework will outline the LEFT considerations at each stage of implementation, detailing what needs to be considered, when, and by or with whom. To guide stakeholders through this roadmap in a more interactive and engaging way, we are incorporating a gamified intervention. This serious game will immerse stakeholders in real-world scenarios, providing them with a hands-on, scenario-based experience as they work through the roadmap's steps. Future research will further refine and validate both the roadmap and the gamified intervention through case studies, ensuring their practical application and contribution to the long-term sustainability of eHealth ecosystems.

### 4.4 Limitations

This study faced several limitations related to the nature of conducting a systematic review of grey literature. Initially, a traditional systematic review methodology was applied, focusing on academic databases. However, naturally these mainly contain peer-reviewed works, which limited the inclusion of true grey literature. To address this, we searched databases specifically geared towards grey literature, such as BASE, NexisUni, and Policy Commons. However, only a few reports met the eligibility criteria, as many lacked relevant insights into the LEFT aspects. Additionally, most of the included documents had already been included in a prior conducted grey literature study (10), suggesting they are considered as standard references in reviews concerning eHealth digitalization and the LEFT factors. However, our study provides added value by specifically focusing on the LEFT factors, a perspective that was underexplored in previous research.

The absence of documents from financial and consultancy firms is another limitation. These sources could provide valuable insights into business models, reimbursement mechanisms, and financial sustainability, which were underrepresented. This may be due to the search strategy's scope or the restricted access to sensitive information. The latter might indicate commercial, political, or confidential concerns, reflecting a larger misalignment of interests between stakeholders, making it more challenging to gather comprehensive data on the subject.

Another limitation was the temporal gap in the included documents, most of which were published between 2020 and 2022. While relevant, these reports do not reflect recent developments in the field, such as the EHDS (8) or the AI Act (34), which could provide critical insights into policy changes and emerging technologies. Moreover, the AI-driven screening method we used (ASReview), effective for academic literature (35), proved less suitable for grey literature due to its differing format, offering no advantage in the screening process. To overcome this, we manually screened the documents and incorporated input from stakeholders with relevant expertise to identify relevant documents. This approach ensured that all documents were selected based on the same eligibility criteria, avoiding automatic biases that could arise. While this manual process ensured consistency in selection, many recommended documents did not align with the study's focus, indicating a mismatch between expert expectations and the study's scope.

While the methodology was designed to address our research aim, the limited relevance of certain recommended documents suggests a need for refinement. A comparative analysis across EU member states could provide valuable insights into best practices and successful strategies in different national contexts. However, it is important to acknowledge that this study did not assess recent stakeholder practices, which may not yet be reflected in published literature. Alternative research methods, such as stakeholder interviews, may help uncover missing insights and ensure a more comprehensive understanding of LEFT aspects, particularly in later stages of eHealth implementation.

## 5 Conclusion

This study underscores the critical importance of understanding the interrelated legal, ethical, financial, and technological (LEFT) challenges in the implementation of eHealth in order to achieve sustainable and effective technologies. While the findings complement several insights from the previous scoping review, they also reveal persistent knowledge gaps, particularly regarding ethical and financial aspects of implementation. Our findings highlight that there is an urgent need for actionable legal and ethical frameworks to simplify compliance and support innovation. Ethical discussions, particularly around consent, responsibility, and accessibility, must be integrated into decision-making at both the governmental and operational levels. Financial infrastructures also require attention, as the lack of business models and fitting reimbursement strategies remain a significant barrier to broader eHealth adoption. Technological barriers, particularly interoperability, cannot be solved by regulatory clarity alone. They demand investment in infrastructure, harmonized data standards, and stronger stakeholder collaboration. Literature indicates a growing awareness of the LEFT barriers and facilitators, yet concrete steps to address them remain scarce, suggesting that misaligned values and priorities hinder coordinated action. However, the complexity, novelty, and dynamic nature of eHealth, alongside the multitude of stakeholders, may be contributing factors to the slow progress in addressing these challenges. To overcome these challenges, it is essential that efforts across all LEFT domains are coordinated. Since obstacles in one domain often affect the others, tackling them in isolation will not lead to sustainable solutions. Moving forward, concrete action must be taken at both European and national levels to address these interconnected barriers. Additionally, continuous monitoring of eHealth implementation across LEFT aspects at the EU level, including the EDHS, is crucial to track progress and ensure alignment with regulatory and societal developments. Only through such integrated efforts can Europe develop robust digital health infrastructures, which build a foundation for interoperable, and trusted eHealth systems that align with evolving technological and regulatory advancements and societal needs.

## Data Availability

The original contributions presented in the study are included in the article/Supplementary Material, further inquiries can be directed to the corresponding author.
